# Does parental involvement matter for children’s academic achievement during school closures?

**Published:** 2022-08

**JULY 7, 2022** - During the COVID-19 pandemic, many children were unable to study in the classroom with teachers’ supervision. A recent study in the British Journal of Educational Psychology reveals an important role for parental involvement in children’s academic achievement during school closures.

In the study of 229 primary school children in China and their parents, the quality of parental involvement as well as the extent of children’s engagement in learning were linked to children’s academic achievement during school closures.

The findings suggested that the most important role in promoting children’s academic achievement was parental support in facilitating children’s autonomous learning. That is, parental involvement facilitated children’s learning engagement, which subsequently improved children’s academic achievement.

Therefore, teachers should encourage parents to give their children educational and psychological support and schools should provide parents with technical guidance and psychological assistance for home-based instructions during school closures.

“As every coin has two sides, parents may make use of the school closure as an opportunity for a closer parent-child relationship, and to promote children’s autonomous academic learning,” said corresponding author Xiujie Yang, PhD, of Beijing Normal University.

 

***Link to Study: https://onlinelibrary.wiley.com/doi/10.1111/bjep.12526***

***Full citation:** “How does parental involvement matter for children’s academic achievement during school closure in primary school?” Xiao Yu, Yinghe Chen, Chunliang Yang, Xiujie Yang, Xin Chen, Xixi Dang, Br. J. Educ. Psychol.; Published Online: 07 July 2022 (DOI: 10.1111/bjep.12526)*.

